# Environmental Source of *Candida dubliniensis*

**DOI:** 10.3201/eid1305.061179

**Published:** 2007-05

**Authors:** Miles A. Nunn, Stefanie M. Schäfer, Michael A. Petrou, Jillian R.M. Brown

**Affiliations:** * National Environment Research Council Centre for Ecology and Hydrology, Oxford, England, United Kingdom; † Hammersmith Hospital National Health Service Trust, London, England, United Kingdom

**Keywords:** fungi, Candida, environment, opportunistic infection, pathogen, dispatch

## Abstract

We isolated *Candida dubliniensis* from a nonhuman source, namely, tick samples from an Irish seabird colony. The species was unambiguously identified by phenotypic and genotypic means. Analysis of the 5.8S rRNA gene showed that the environmental isolates belong to *C. dubliniensis* genotype 1.

The ever increasing number of immunosuppressed humans has led to a marked rise in opportunistic infections, particularly those caused by fungi (*1*). *Candida albicans* is the yeast species most commonly associated with oropharyngeal and systemic candidiasis in immunocompromised persons. However, the last 2 decades have seen an increase in infections by other *Candida* species, including *C. dubliniensis,* which was first recognized as distinct from *C. albicans* in 1995 in Ireland (*2*,*3*). *C. dubliniensis* has been recovered mainly from the oral cavities of HIV-infected persons (*4*) but also from lungs, vaginas, blood, and feces; occasionally this organism causes fatal systemic infections (*5*). Isolates are assigned to 4 genotypes, defined by the sequence of the internal transcribed spacer regions of the rRNA gene (*6*).

*C. dubliniensis* is globally distributed. In HIV-infected patients, the oral prevalence is 1.5%–32% (*5*). In healthy persons not infected with HIV, *C. dubliniensis* is absent or rare, but 14% of healthy Caucasians had oral *C. dubliniensis* in a South African study (*7*). Like *C. albicans*, *C. dubliniensis* may be a member of the normal oral microbial flora of humans, and oral candidosis may result from overgrowth of resident strains. In contrast to other *Candida* species, some of which are associated with birds (*8*,*9*), *C. dubliniensis* has not been found to date in nonhuman environmental sources. This has led to speculation that the species may be restricted to humans, possibly occupying sites deep within the oropharynx or upper respiratory tract (*5*).

## The Study

Fungal strains were obtained from *Ixodes uriae* ticks (as part of a National Environment Research Council–funded study of a tickborne virus) at a seabird breeding colony on Great Saltee Island, Ireland (52°07′N, 6°36′W). The ticks were taken from cracks in cliffs used by common guillemots (*Uria aalge*). Tissue cultures of tick homogenates undertaken for virus isolation were occasionally contaminated with yeastlike fungi.

To investigate this, individual adult ticks were homogenized in 1 mL minimum essential medium (MEM). After centrifugation (30 s, 10,000× *g*), 0.2 mL of supernatant was added to 4 mL MEM, 5% fetal bovine serum, and 100 μg/mL penicillin-streptomycin. Cultures incubated at 37°C were examined microscopically daily for up to 6 days. Positive cultures were plated twice on Sabouraud dextrose agar (SAB) with chloramphenicol (BioMérieux, Marcy l’Etoile, France) before phenotypic testing. Isolates were identified by using API identification kits (BioMérieux) and by conventional methods (*10*). Antifungal drug susceptibility was tested according to the Clinical Laboratory Standards Institute guidelines (*11*). The control strains were *C*. *albicans* ATCC 90028, *C. glabrata* ATCC 90030, *C*. *krusei* ATCC 6258, and *C. dubliniensis* NCPF 3949.

Internal transcribed spacer 1 and 2 regions (ITS1/ITS2) and the 5.8S rRNA gene were amplified with primers ITS1 and ITS4, described by White et al. (*12*). Template DNA was prepared by boiling single SAB-grown colonies in 50 μL ultrapure water for 10 min. After centrifugation (5 min 10,000× *g*), 15 μL supernatant was added to 50 μL PCRs containing 1× reaction buffer, 1 μmol/L ITS primers, 1.5 mmol/L MgCl_2_, 400 μmol/L deoxynucleoside triphosphates, and 2.5 U Immolase (BiolineLtd, London, England, UK). Cycling parameters were 7 min 95°C, 30 cycles 1 min 95°C, 1 min 58°C, 1 min 72°C, and a final extension of 5 min 72°C. Products were purified (QiaQuick kit, QIAGEN Ltd., West Sussex, England, UK) and sequenced (BigDye kit and ABI 377 sequencer; Applied Biosystems, Foster City, CA, USA) by using the ITS primers. Sequences were assembled by using Lasergene 6, Seqman version II, and aligned by using BioEdit software (*13*).

Fungal isolation was undertaken on 2 separate days with samples from 2 distinct sites on the island (Table 1). On both days, Happy Hole West (HHW) ticks were processed immediately after Labour in Vain (LIV) ticks in the same class II microbiologic cabinet. No fungi were detected in HHW ticks, whereas 16.7%–27.6% of ticks sampled from 2 locations within LIV gave positive cultures (Table 1). Twenty-two isolates were obtained (Table 1); SL370–429 were from LIV-1 and SL495–531 from LIV-2 (SL = Saltee).

**Table 1:** Male and female ticks positive for fungi in culture*

Site	No positive/no. examined		
	Male	Female	Both sexes
Happy Hole West (HHW)–1	0/26	0/25	0/51
HHW-2	0/17	0/23	0/40
Labour in Vain (LIV)–1	5/30	8/29	13/59†
LIV-2	5/23	4/20	9/43‡

*Adult *Ixodes uriae* ticks were collected from within 2 guillemot-breeding colonies on Great Saltee on August 25, 2004. The ticks were stored frozen and processed by M.A.N on November 11, 2005. (HHW-1 and LIV-1) and 20. 07.06 (HHW-2 and LIV-2).

†Isolates SL370, SL371, SL375, SL387, SL397, SL407, SL410, SL411, SL413, SL414, SL417, SL422, and SL429.

‡Isolates SL495, SL497, SL500, SL501, SL509, SL510, SL522, SL529, and SL531.

**Figure 1:**
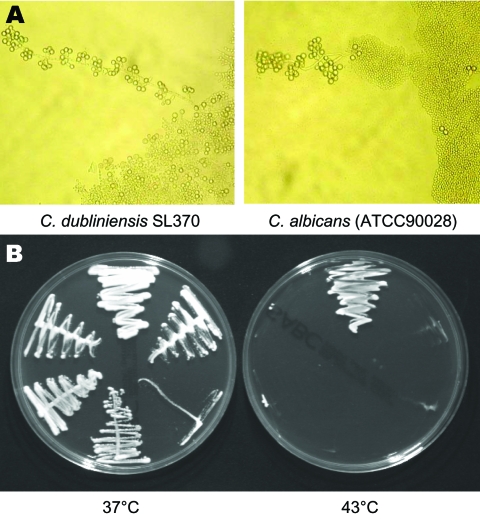
Phenotypic characteristics of environmental *Candida dubliniensis* isolates and reference strain of *C. albicans*. A) Morphology of pseudohyphal terminal chlamydospores of *C. albicans* (ATCC90028) and *C. dubliniensis* SL370 grown at 37°C on Corn Meal Tween 80 agar. Magnification × 50. B) Growth of representative Great Saltee (SL) isolates on Sabouraud agar after 48 h of incubation at 37°C and 43°C. The growth of the following isolates is shown: *C. albicans* (ATCC90028), *C. dubliniensis* (NCPF3949), and *C. dubliniensis* SL370, SL397, SL407, and SL410 (clockwise from the top in each panel).

**Figure 2:**
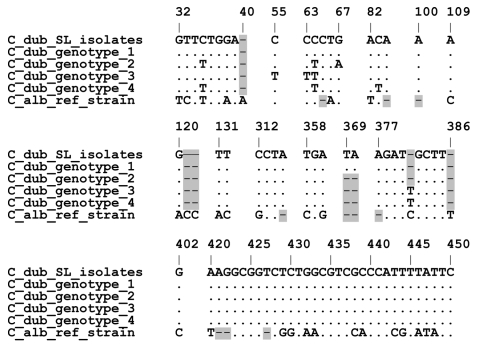
Alignment of the ITS1 5.8S ITS2 DNA region of *Candida dubliniensis* Saltee (SL) isolates and representative human isolates of genotypes 1–4. All polymorphic sites are shown: ITS1 (bp 32–137), 5.8S rRNA exon (bp 312–315), ITS2 (bp 358–450). Dots indicate identity; highlighted dashes indicate gaps in the alignment. GenBank accession nos. are as follows: AJ311895 (CD33 genotype 1), AJ311896 (CD520 genotype 2), AJ311897 (CD519 genotype 3); AJ311898 (p7718 genotype 4), AB049119 (*C. albicans* ATCC90028), and EF032487–EF032495 for SL375–1, SL397, SL375–2, SL387, SL407, SL410, SL411, SL417, SL422, respectively.

On SAB the colonies from positive cultures were a creamy white color with a glabrous appearance similar to *C. albicans*. SL375 had a mixed phenotype (large and small colonies, designated SL375–1 and SL375–2). Like *C. albicans*, all SL isolates were germ-tube positive and produced chlamydospores at 37°C on Corn Meal Tween 80 agar (Oxoid Ltd, Basingstoke, England, UK) and Czapek Dox (1%) Tween 80 agar (Oxoid) (Figure 1A). None of the SL isolates grew at 43°C on SAB (Figure 1B), which suggests that they might be *C. dubliniensis* (*14*). This was confirmed by carbohydrate assimilation tests (Table 2) and by sequencing the 5.8S rRNA gene (Figure 2). With the API 20C AUX kit, all SL isolates yielded the same profile at 48 h, interpreted as 99.9% *C. dubliniensis* (Table 2). Eleven isolates from LIV-1 and the *C. dubliniensis* (NCPF 3949) reference strain were also tested with API 32C. All had an identical profile (7143100015), interpreted as 81.9% *C. dubliniensis* and 16.9% *C. albicans*.

**Table 2:** Substrate assimilation by Great Saltee fungi and *Candida albicans*

Substrate	API 20C AUX assimilation profile code*	
	SL407	*C. albicans* (ATCC90028)
Pentoses		
L-arabinose	–	–
D-xylose	–	+
Hexoses		
D-glucose	+	+
D-galactose	+	+
α-methyl-D-glucoside	–	+
Disaccharides		
D-cellobiose	–	–
D-lactose	–	–
D-maltose	+	+
D-saccharose	+	+
D-trehalose†	–	+
Trisaccharides		
D-melezitose	–	–
D-raffinose	–	–
Alcohols		
Glycerol	+	–
Adonitol	+	+
Xylitol	+	+
Inositol	–	–
D-sorbitol	+	+
Organic acids		
2-keto-gluconate	+	+
Amino acids		
N-acetylglucosamine	+	+
		
Identification	*C. dubliniensis*	*C. albicans*
API 20C AUX profile code	6172134	2566174
Predictive value	99.9%, excellent	99.2%, very good

*Results for 48 hours are shown. All 22 Great Saltee isolates gave similar profiles.

†By 72 hours all Saltee isolates showed some assimilation of trehalose; the degree of assimilation varied between isolates.

ITS sequences of isolates SL375, SL397, SL407, SL410, SL411, SL417, and SL422 were identical to that of *C. dubliniensis* CD33 genotype 1 (Figure 2). Nevertheless, phenotypic variation among the SL isolate was evident. In addition to variation in trehalose assimilation rates, 3–4 distinct types were apparent in the germ tube test. Three independent inoculations (10^5^ CFU/mL, mid log growth phase) of each isolate gave consistent morphologic differences. One group produced very long germ tubes (SL375–1, SL422, SL417 SL387, SL397, and SL413); another, medium size (SL411, SL407); a third, mainly long germ tubes with cells that aggregate like *C. parapsilosis* (SL375–2 and SL410); and the last, elongated cigarlike cells that clamped together with few germ tubes (SL370 and SL371).

To determine whether *C. dubliniensis* was present on their outer surface LIV-2 and HHW-2 ticks (n = 102) were individually washed in 1 mL MEM before homogenization. *C. dubliniensis* was cultured from the wash supernatants of 8 of 9 ticks that proved positive after homogenization but not from any of the negative ticks. These findings strongly suggest that the fungus is present in particles adhering to the ticks.

The SL isolates (n = 11) were extremely sensitive to antifungals. MIC90s (μg/mL) were as follows: amphotericin B 0.031 (Sigma-Aldrich, Saint Louis, MO, USA), flucytosine 0.031 (Valeant Pharmaceuticals International, Aliso Viejo, CA, USA), fluconazole 0.125 (Pfizer Inc., Sandwhich, Kent, England, UK), itraconazole 0.007 (Ortho Biotech, Bridgewater, NJ, USA), voriconazole 0.007 (Pfizer), posaconazole 0.007 (Schering-Plough, Kenilworth, NJ, USA), ketoconazole <0.007 (Ortho Biotech), and caspofungin <0.007 (Merck & Co., Inc., Rahway, NJ, USA).

## Conclusions

Our serendipitous isolation of *C. dubliniensis* from the environment ends speculation (*5*) that the species might be confined to humans. Because ticks were collected from cracks filled with guillemot guano and the fungal isolates were associated with the surface of ticks, the most likely source of the fungus is bird excrement. Thus, like *C. albicans* (*8*), *C. dubliniensis* may inhabit the digestive tract of marine birds. Only *C. dubliniensis* was isolated from the Great Saltee ticks; however, we have detected other *Candida* spp. from both soil and tick samples (unpub. data) at other seabird colonies, which suggests that marine as well as terrestrial birds (*9*) carry a variety of yeast species (*15*).

The environmental isolates are members of human *C. dubliniensis* genotype 1, commonly associated with HIV patients (*6*). Sequencing of the genotype 1 CD36 isolated from an HIV patient is nearly complete (www.sanger.ac.uk/sequencing/Candida/dubliniensis). Phylogenetic comparison of variable loci from environmental and human genotype 1 isolates could be used to estimate when they last shared a common ancestor and thus how often *C. dubliniensis* is transmitted from the environment to humans.
